# Bending Performance of Reinforced Concrete Beams with Rubber as Form of Fiber from Waste Tires

**DOI:** 10.3390/ma17204958

**Published:** 2024-10-11

**Authors:** Ali Serdar Ecemiş, Emrah Madenci, Memduh Karalar, Sabry Fayed, Essam Althaqafi, Yasin Onuralp Özkılıç

**Affiliations:** 1 Department of Civil Engineering, Necmettin Erbakan University, 42090 Konya, Türkiye; 2 Science and Technology Research and Application Center (BITAM), Necmettin Erbakan University, 42090 Konya, Türkiye; 3 Department of Technical Sciences, Western Caspian University, Baku 1001, Azerbaijan; 4 Department of Civil Engineering, Zonguldak Bulent Ecevit University, 67100 Zonguldak, Türkiye; 5 Department of Civil Engineering, Faculty of Engineering, Kafrelsheikh University, Kafrelsheikh 6860404, Egypt; 6 Civil Engineering Department, College of Engineering, King Khalid University, Abha 61421, Saudi Arabia; 7 Department of Civil Engineering, Lebanese American University, Byblos 03797751, Lebanon

**Keywords:** reinforced concrete, beam, waste, rubber, recycled

## Abstract

An investigation was conducted to assess the efficacy of using waste rubber as a substitute for a portion of an aggregate to enhance concrete’s sustainability. For the purpose of accomplishing this objective, a total of 12 specimens were constructed and then subjected to a series of tests to investigate their bending behavior. The samples were constructed with the following dimensions: 1000 mm length and a 100 mm by 150 mm cross-sectional area. A few factors were selected, including the impacts of the longitudinal reinforcement ratio and the waste rubber ratio. Based on the volume of aggregates, rubber replacement rates of 0%, 5%, 10%, and 15% were investigated in this study. To assess the beam bending behavior, the stirrup width and spacing were kept constant at ∅6/10. The longitudinal reinforcement was composed of three diameters: ∅6 at the top (for all beams) and ∅8, ∅10, and ∅12 at the bottom. The experimental results demonstrated that the effects of varying amounts of waste rubber and tension reinforcement on the bending and cracking of reinforced concrete beams (RCBs) were varied. The findings indicate that the incorporation of waste rubber into concrete results in a reduction in both the load-carrying capacity and the level of deformation of the material. Additionally, it was shown that as the amount of waste rubber in the RCB increased, the energy absorption capacity and ultimate load decreased. There was a reduction in energy dissipation of 53.71%, 51.69%, and 40.55% for ∅8 when longitudinal reinforcement was applied at 5%, 10%, and 15% replacement, respectively. Additionally, there were reductions of 25.35%, 9.31%, and 58.15% for ∅10, and 38.69%, 57.79%, and 62.44% for ∅12, respectively.

## 1. Introduction

Every year, automobiles in both industrialized and developing nations produce millions of used tires, and it has become a serious environmental problem [1,2]. A significant number of the 1.4 billion tires sold globally each year eventually fall into the category of End of Tires [1]. The US Environmental Protection Agency (EPA, 2007) reported that approximately 290 million waste tires were generated in 2003. Out of these, 45 million were recycled into new car and truck tires. In Europe, 90 facilities produce 355 million tires annually, accounting for 24% of global tire production [3]. Malaysia produced 38,200 tons of garbage per day in 2016 (with a 17.5% recycling rate), with scrap tires accounting for a large portion of the waste. Over 30% of possibly recyclable products, such as plastic and used tires, are still dumped in landfills due to improper garbage sorting [4]. Recycling waste tire rubber has become a worldwide issue, especially in China, where the yearly production of scrap tires exceeds 15 million tons [5]. One of the most important issues facing municipal governments nowadays is how to properly handle and dispose of a large amount of discarded tires [6,7]. Scientists have investigated the utilization of recycled crumb rubber derived from discarded tires to partially substitute for natural aggregates in concrete, thereby advancing the circular economy concept in the field of building [8,9]. Furthermore, waste tires can be used in various construction materials such as asphalt, culverts, bricks, paving stones, and acoustic panels. They can also be used as mulch in sports surfaces, as a binder modifier in asphalt, and in geotechnical applications such as road and embankment backfills [10]. Compared with conventional concrete, rubberized concrete (RuC) possesses many benefits, including increased plastic energy capacity, damping, ductility, and resistance to freeze–thaw cycles [11,12,13,14,15]. On the other hand, when rubber aggregates are used in place of all-natural aggregates, the cement matrix link is weakened, leading to a reduction in compressive and flexural strength by up to 80% and 35%, respectively [14,15,16].

The use of wasted rubber as an aggregate replacement in concrete has increased recently [17,18]. Abdelmonem et al. [19] examined how rubber incorporation affects the mechanical characteristics of high-strength concrete. The results demonstrate substantial decreases in the mechanical characteristics of concrete with higher rubber content. The structural weakness can be explained by the propensity of the cement slurry to crack around low-hardness rubber particles when subjected to loading [19]. A study was carried out by Karunarathna et al. [20] to investigate the effects of two distinct rubber aggregate particle sizes on the mechanical properties of concrete. Based on the results, Karunarathna et al. [20] concluded that concrete’s elastic modulus and compressive strength (CS) decrease when rubber aggregate’s modulus decreases. On the other hand, the bonding interface between cement paste and rubber particles significantly affects the mechanical properties of reinforced concrete. Additionally, it was noted that the decrease in the mechanical properties of reinforced concrete is less significant when the rubber particles used are smaller in size [20]. Roychand et al. [8] emphasized the importance of the rubber particle size in affecting the mechanical characteristics of concrete. The results also demonstrate that an increase in rubber smoothness leads to an increase in concrete’s technical qualities independent of replacement amount. Conversely, some studies have explored different methods to counterbalance the reduction in the engineering characteristics of concrete, including rubber additives. According to Chou et al. [21], the mechanical properties of materials are improved when rubber particles are exposed to an alkaline solution before being included in concrete. Han et al. [22] examined the stress–strain properties of RuC after incorporating waste-tire crumb rubber. Their study aimed to address the lack of established rules for designing rubberized concrete. The study examined the ways in which crumb rubber affects the stress–strain behavior of concrete, considering aspects such as differences in rubber content and pre-treatment procedures. Notable changes were observed, such as a discernible decrease in strength as the rubber component increased. The study found that the addition of crumb rubber had a substantial impact on the coordination of stress and strain in concrete, which in turn affected its stress–strain behavior. Mohammed and Ali [23] examined the flexural characteristics of rubberized reactive powder concrete beams incorporating different amounts and dimensions of discarded tire rubber and compared them to the flexural behavior of conventional reactive powder concrete. To achieve this objective, a total of 13 beams were tested at two loading sites. The testing involved applying repeated loads to small beams measuring 1100 mm in length, 150 mm in width, and 100 mm in height. The amount of crumb rubber substituted for the fine aggregate was 5, 10, and 15%, respectively. The silica fume was replaced at percentages of 10, 20, 30, and 50% using extremely fine rubber. The incorporation of discarded rubber and steel fibers resulted in a reduction in fracture width. The shear behavior of concrete beams made of polypropylene fiber, recycled concrete aggregate, and crumb rubber was investigated by Hossain et al. [24]. To achieve this objective, a set of 15 reinforced concrete beams was fabricated using varying proportions of recycled concrete aggregate (ranging from 0% to 50%), crumb rubber as a fine aggregate (ranging from 0% to 10%), and polypropylene fiber fractions (ranging from 0% to 1%). The experimental results indicate that incorporating crumb rubber into the concrete mixture has a detrimental impact on the beam’s ultimate shear strength, resistance to diagonal cracking, toughness, and deformability. By adding a small amount of rubber (up to 5%), the beam deformability is enhanced. Al-Hajjar and Khafaji [25] experimentally studied the impact behavior of RuC beams. Predicting the maximum mid-span movement and impact load of the RuC beams was the goal of this investigation. Under impact load testing, four RCBs with different rubber contents (0%, 25%, and 35%) were tested at heights of 1.5, 2.0, and 2.4 m. The findings indicate that adding RuC leads to a decrease in the maximum impact force by 2.93%, 13.16%, and 17.53% at rubber contents of 15%, 25%, and 35%, respectively, compared to the impact force without RuC. The beams with 15% and 25% replacement ratios decreased displacement by 8.44% and 6.26%, respectively, at the 2.4 m mass drop height, whereas the beam with 35% rubber content increased displacement by 17.26% compared to the reference beam. Kadhim and Kadhim [26] observed how the amount of rubber used to replace natural aggregates, the size effect, and the shear span-to-overall height ratio affected the overall shear load capability. As an alternative to coarse aggregates (10–4.75 mm size) and fine aggregates (4.75–0.15 mm size), the rubbers used had varying volumetric proportions, specifically 5%, 10%, 15%, and 20%. In continuous deep beams, the 20% volumetric replacement of natural coarse or fine aggregates with tier rubber decreased the ultimate load by 32.06% and 32.65%, respectively, but increased the ultimate deflection by 83.07% and 106.28%. Another investigation was performed by Jayanath et al. [27]. An investigation into the behavior of reinforced RuC beams under flexure was carried out through a series of four-point bending tests. The results of the flexural test revealed that the RuC beams recovered after unloading and exhibited larger deflections than regular concrete. Sharaky et al. [28] performed another investigation. The investigation involved testing nine RCBs with functionally graded materials and plain concrete to enhance the positive impacts of crumb rubber and minimize its negative impacts on beam performance. In the center layer of the beam section, as the amount of crumb rubber increased, the beam stiffness dropped slightly until it reached 50% crumb rubber content. At 80% crumb rubber content, the beam stiffness decreased further. Karalar et al. [29] investigated the impact of waste tire rubber on the flexural behavior of RCBs. Waste-tire rubber volumetric proportions were chosen to range from 0% to 7.5% across the entire concrete mixture to achieve this objective. According to observations, the ability of RCBs to bend and rupture is affected differently by each waste tire rubber that is combined with them. Eisa et al. [30] conducted another investigation to observe the influence of a mixture of crumb rubber and steel fibers on the structural response of RCBs subjected to static loads. At several weight percentages (5%, 10%, 15%, and 20%), crumb rubber (size 2–3 mm) was used to partially substitute fine aggregates. Upon the conclusion of this experiment, it was determined that the augmentation of the rubber content by up to 10% in RCBs enhanced their resistance to cracking and increased their ability to deform without fracturing. Alasmari et al. [31] examined the impact of RuC in a hybrid form consisting of a top layer composed entirely of RuC and a bottom layer composed of regular concrete with fiber. Eight beams, each measuring 150 × 200 mm in cross-section and 1000 mm in length, were experimentally tested over an effective span of 900 mm. Further, 10%, 12.5%, and 15% volumes of crumb-rubber aggregate were substituted for the sand river. Hybrid structure modeling demonstrated superior performance for various features compared to the controlled model without additional materials. According to the information presented above, many studies have been conducted in the literature concerning the performance of concrete with waste rubber tires [32,33,34,35,36,37,38,39]. The findings of a comprehensive study on the important properties of rubberized components make it abundantly clear that waste rubber tire aggregates can serve as partial substitutes for both coarse and fine aggregates. Furthermore, new developments have shown that more successful mechanical results can be obtained when tires are pre-treated. For example, when tires smaller than 0.6 mm in size were used and treated with NaOH + CSBR Latex + SCA, very close compressive and flexural strength values were achieved with the reference sample even at 5%-10%-15% tire substitution values [40]. Similarly, when tires with dimensions smaller than 1.18 mm were used, compressive strength values higher than the reference specimen were obtained at a 10% tire replacement rate when cured with both methanol and acetone [41]. When 3.0 mm sized tires were used and cured with CS2, a 10% and 20% increase in RuC compressive strength was obtained at 3% and 6% tire replacement rates, respectively, compared with the reference specimens [42]. Considering the flexural strength, when waste tires with dimensions smaller than 6 mm were used and pre-coated with cement paste and cement mortar, increases of 7% and 10% in the flexural strength compared to the reference specimen were obtained with a 38% tire replacement rate, respectively [43]. A 77% increase in flexural strength was obtained when using very small (<500 μm) waste tires and pre-treating with NaOH, water washing, and a 10% mass of the cement paste [44]. When RuC was produced using waste tires with sizes ranging from 0.6 to 2.5 mm with a 10% replacement rate after pre-treatment with saturated NaOH solution, 32% sulfuric acid solution, and saturated Ca(OH)_2_ solution, increases of 2%, 10%, and 5% in flexural strength were obtained compared with the reference samples, respectively [45].

Although there are studies in the literature on the use of waste tires as aggregates in concrete and the effect of waste tires on the mechanical properties of concrete, no research has examined the flexural behavior of reinforced concrete considering tire shape. In this study, the flexural behavior of a reinforced concrete beam with different longitudinal reinforcement ratios using different ratios of specially fiber-formed waste tires was investigated. The details of the study are provided in the following sections.

### The Objective of the Study

An analysis of the available literature reveals that the utilization of waste rubber has been prevalent in most of the limited research studies on reinforced concrete. Nevertheless, studies on the bending behavior of reinforced concrete with waste rubber as the material component have not yet been published. Based on these observations, we observed that the structural performance of beams constructed from concrete reinforced with waste rubber has not been the focus of any current study. One of the most important aspects of the bending problem in RCBs is the evaluation of the interactions between different waste rubber ratios and concrete. This investigation is significant because it examines these interactions. This study aims to investigate the strengthening capabilities of concrete in beams to advance our knowledge of concrete and various waste rubber ratios. This process is accomplished by substituting aggregates for traditional components in the production of concrete using different waste rubber ratios. A total of 12 beam specimens with constant stirrup spacing were manufactured and evaluated. The parameters examined in this investigation are rubber replacement rates of 0%, 5%, 10%, and 15%, as determined by the volume of the fine and coarse aggregates. To improve the usage of used tires as an environmentally friendly building material, an analysis of the experiment’s load deflection curves was conducted.

## 2. Experimental Investigation

### 2.1. Characteristics of Rubberized Concrete (RuC)

**Figure 1:**
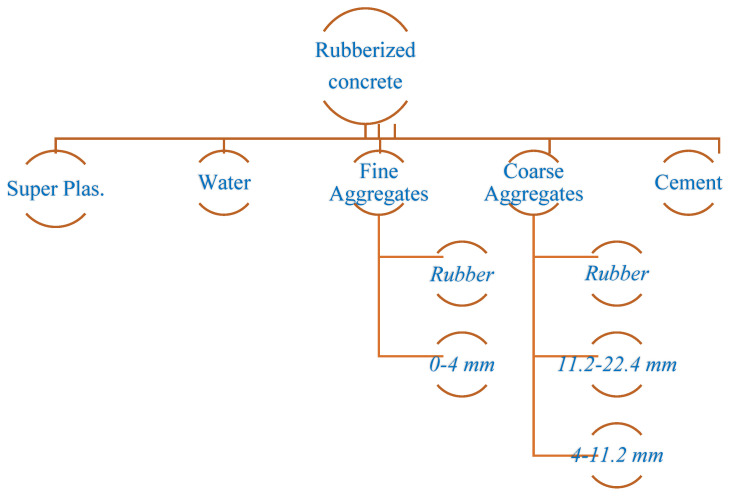
Rubberized concrete mixture.

**Figure 2:**
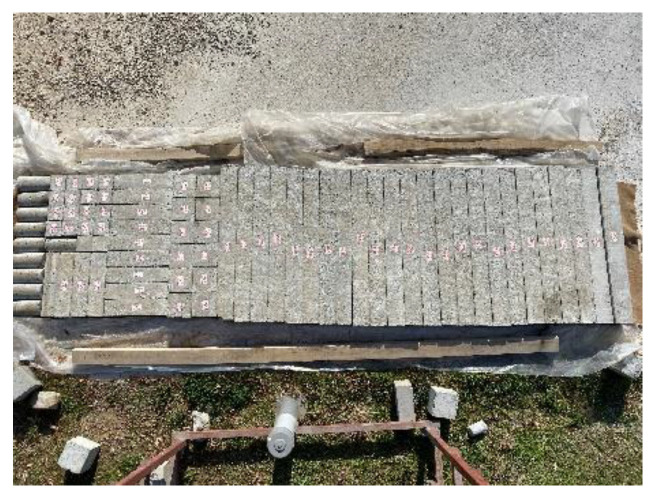
Arranged and categorized the test specimens.

The experimental program of this study is the same as that of a previously published paper, which is the first part of this study [46]. In the first part of the study, using the same mixing method, concrete design and rubber ratios, the effect of the waste rubber ratio in fiber form on the shear behavior of RuC beams was investigated. Similarly, a total of twelve reinforced concrete beam specimens of 100 mm × 150 mm × 1000 mm (g × d × L) each were fabricated and tested considering different volumetric stirrup ratios of 2.10, 2.80 and 3.53. In this study, different longitudinal reinforcement ratios were used. The volumetric proportions of waste rubber in fiber formation were estimated to be 5%, 10%, and 15%. Figure 1 shows the methods used in this study. Furthermore, Table 1 lists the ratios for mixing concrete, and Figure 2 shows the manufactured test specimens.

**Figure 3:**
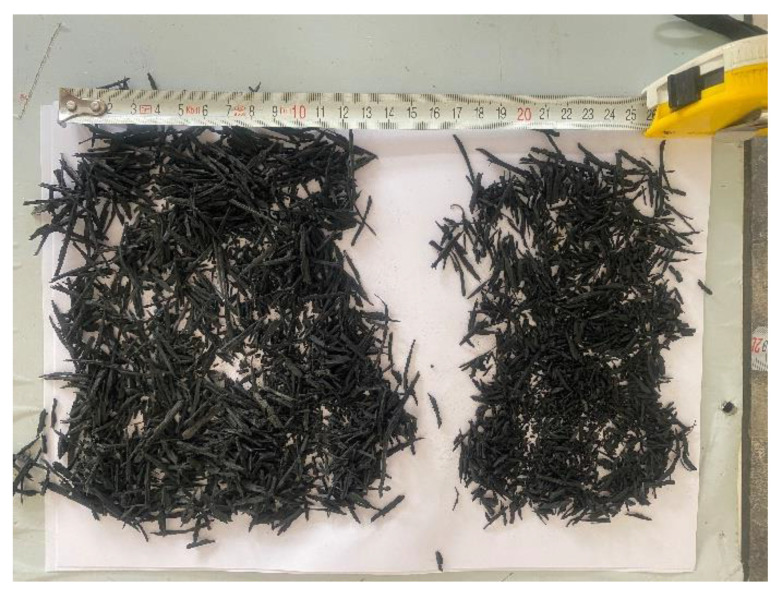
Waste tire rubber in the form of fiber.

Aggregate obtained from the Konya region was used in this study. Two distinct types of coarse aggregates (4–11.2 mm and 11.2–22.4 mm) and fine aggregates (0–4 mm) were used. Furthermore, waste tire sizes in the formation of fiber were chosen to be in two distinct categories: fine and coarse. Figure 3 displays the sizes of the waste tires used in the investigation. The flexural strength of concrete when fiber-formed rubber is used is higher than the flexural strength of concrete when crumb rubber is used. On the other hand, when the compressive strength is considered, the compressive strength decreases when crumb rubber is used. The obtained results varied with the tire size as well as the shape of the used tire. In addition, there are many studies in the literature where tires are subjected to pre-treatment to increase their mechanical properties and eliminate the negative effects of the tire.

When crumb rubber is used, the size effect becomes more pronounced. In the compressive strength tests performed on two different RuC specimens with tire replacement rates of 10%, 15%, and 20% and tire sizes of <0.3 mm and 4–10 mm, the results obtained in RuCs using <0.3 mm waste tires were 73.4%, 73%, 67.3%, 69%, 46%, 34.5%, respectively, compared to the reference specimen, while the results obtained in the specimens produced using waste tires with 4–10 mm dimensions were 69%, 46%, and 34.5% [47,48].

The CEM I 42.5 R-type cement (Konya Cement, Selçuklu, Türkiye). Table 2 lists the characteristics of the cement manufactured in compliance with TS-EN-197-1 [49].

**Figure 4:**
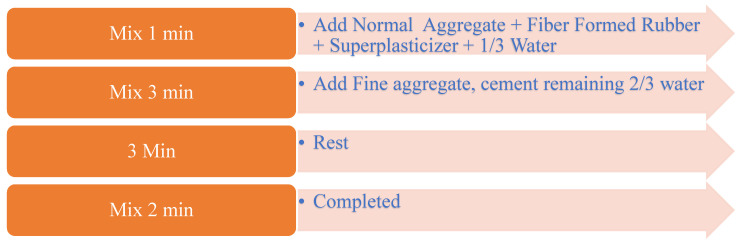
RuC production phases.

During the preparation of the mixture, normal aggregates and lightweight aggregates obtained from fiber-formed waste tires (if any) were placed in the mixer together with 1/3 of water and superplasticizer. After mixing for 1 min, fine aggregate, cement, and the remaining 2/3 of water were added to the mixture and mixed for another 3 min. After mixing for 3 min, the mixture was left to rest for 3 min. After 3 min of resting, the mixture was mixed for another 2 min and the RuC production process was completed. During all mixing processes, special care was taken to avoid segregation. The concrete production stages are illustrated in Figure 4.

**Figure 5:**
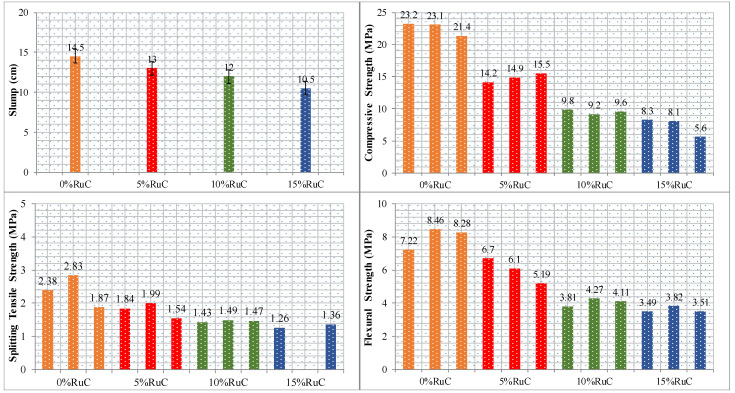
Results of the mixture’s 28-day test.

In this investigation, Politan 612—a superplasticizer concrete addition based on polycarboxylate that lowers the mixing water of concrete, boosts all strengths, and enhances the workability of fresh concrete—was utilized. The ASTM C 494 [50] and TS EN 934-2 [51] standards provide limit values on the impact of this superplasticizer on concrete performance that are in line with these limits. The 28-day CS testing results for the mixture are presented in Figure 5. Ultimately, a greater percentage of waste rubber had a negative effect on the CS of RuC. Increasing the Ru percentage from 0% to 15% resulted in a decrease in CS of 34.2%, 57.7%, and 67.5%, respectively, after 28 days, which are the changes obtained compared with 0% RuC. The decrease in strength can be attributed to the interfacial transition zone (ITZ) between the aggregate and mortar in the concrete. This zone is commonly considered the weakest component of the concrete composite structure due to the wall effect [52] and the accumulation of water at the aggregate surface. This leads to a high water-to-cement ratio caused by bleeding [53]. Consequently, the presence of enhanced porosity and local weakness at the ITZ increases the material susceptibility to microcracking under stress. Owing to the insufficient connection between the rubber and mortar, this problem is more noticeable when RuC is used. This fragile contact facilitates the formation of supplementary microcracks at the periphery of the rubber particles [54,55,56,57]. In addition, the moduli of elasticity of cement mortar and rubber granules are distinct from one another in a significant way. Under loading conditions, this disparity causes an increase in tensile stress at the contact points, which propagates tiny cracks and leads to the formation of additional fractures [55]. Additionally, earlier experiments revealed that the measurement of air content improved in proportion to the amount of Ru present. The mixture’s mechanical characteristics may have suffered as a result, leading to an increase in porosity [54]. Tensile strength measurements in split tests frequently exhibit a pattern that resembles the CS of concrete. The split tensile strength decreased in direct proportion to the increase in Ru replacement. The split tensile strength experienced a decrease of 24.2%, 38.1%, and 44.5%, while the fraction of Ru increased from 0% to 5%, 10%, and 15%, respectively. The same reason for this reduction may have contributed to the decline in concrete’s CS as the percentage of Ru increased. A 25.0%, 49.2%, and 54.8% decrease in the flexural strength was observed as the proportion of Ru was progressively increased from 0% to 5%, 10%, and 15%. The reasons behind the decline in CS that coincided with the increase in the Ru content may also be responsible for this decline. The results of the mixture’s 28-day test are given in Figure 5.

### 2.2. Experimental Configuration

**Figure 6:**
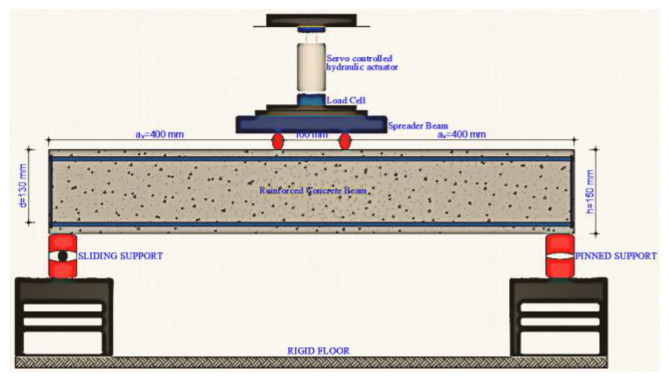
Experimental configuration.

The beam specimens were subjected to flexural tests using a four-point loading configuration. Two types of support, fixed and hinged, were used in the experimental setting. Throughout the experiment, the load and displacement data were noted, and each phase was documented using photos and videos. Figure 6 illustrates the experimental setup.

Four distinct mixes, including the reference mix, were used in the experimental concrete composition. The Necmettin Erbakan University Civil Engineering Laboratory conducted an investigation to examine the bending performance of reinforced concrete beams. To achieve this, 12 beam specimens were created using constant stirrup spacing and variable ratios of fibers generated from waste rubber as aggregates. A width of 100 mm, height of 150 mm, and length of 1000 mm were determined as the dimensions of the beam specimens. The experiments were conducted with beams identical in size to one another. Therefore, to evaluate the bending behavior of the RCBs, the stirrup diameter and spacing were maintained at a constant value of ∅6/10. The three diameters that comprise the longitudinal reinforcement were ∅6 at the top (in all beams) and ∅8, ∅10, and ∅12 at the bottom. The beam reinforcement ratio (δ = 0.00125) was selected to be less than the balanced reinforcement proportion to achieve ductile behavior in all beams. Three different waste tire ratios (W_ru_) of 5%, 10%, and 15% were used to manufacture the test specimens. Table 3 lists the characteristics of the samples.

## 3. Test Results

This section of the study clearly and thoroughly presents and evaluates the impact of varying levels of tension reinforcement and waste rubber on the deformation and bending behavior of RCBs. Therefore, the laboratory-produced RCBs were tested for fracture and bending behavior. As previously mentioned, a total of 12 test samples were prepared for this purpose. Following the preparation of 12 distinct RCBs in the laboratory—these specimens were next subjected to fracture and bending tests. Each quantity of waste rubber had a different impact on the RCBs in terms of bending and fracture, as demonstrated by the detailed estimation of the RCB fractures. In addition, throughout the research, each RCB exhibited distinct bending characteristics depending on the amount of waste rubber present. Regarding the examination of the impact of the waste rubber quantity on the fracture and bending attitude of concrete structures, it is very important to consider that each RCB has a unique capacity for carrying loads. As shown in Table 4, 12 distinct RCBs were examined in this investigation.

### 3.1. Effect of Tensile Reinforcement Proportions on Waste Rubber Concrete Beams (RuCBs) 

**Figure 7:**
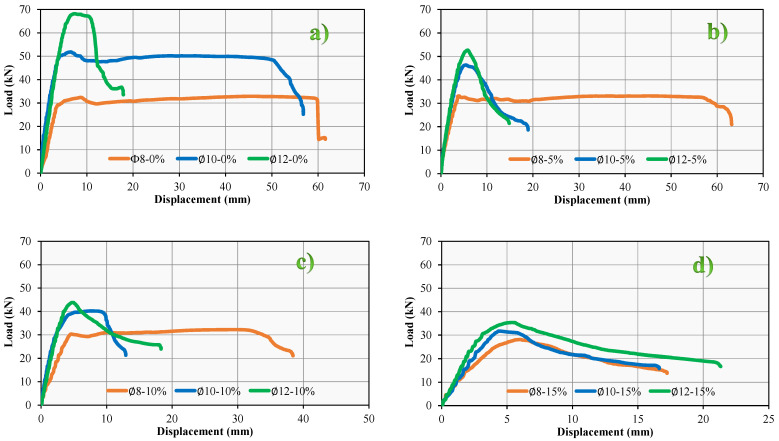
RCB load–deformation attitude for (**a**) ∅8-0%, ∅10-0%, ∅12-0%, (**b**) ∅8-5%, ∅10-5%, ∅12-5%, (**c**) ∅8-10%, ∅10-10%, ∅12-10%, (**d**) ∅8-15%, ∅10-15%, ∅12-15%.

**Figure 8:**
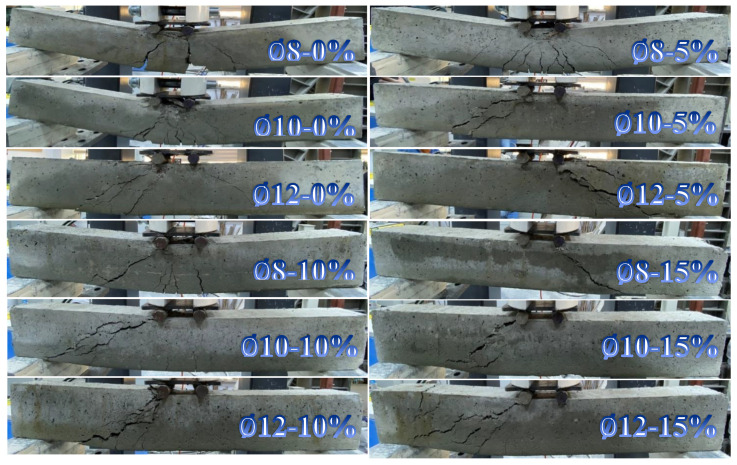
The ability of the RCB to break and bend.

It was observed that the reference RCB exhibited significant bending cracks that were dependent on the vertical load. This was a result of the experimental results that were provided. The LVDT device was used to measure the maximum deflection in the RCBs under vertical load. These bending measurements are clearly depicted in Figure 7. According to the data presented in Figure 7, the degrees of bending steadily increased in a linear fashion until reaching their highest points. As shown in Figure 8, RCBs and RuCBs can develop significant fractures when subjected to vertical load. Furthermore, bending cracks of noteworthy magnitude are shown in Figure 8. These cracks are the locations in the RCBs and RuCBs where vertical cracks can occur. Values of 32.37, 51.82, and 68.19 kN for ∅-0%, ∅10-0%, ∅12-0%, respectively, were determined at the end of this straight line. At the maximum vertical load, deflections of 8.75, 6.45, and 7.37 cm are reported for ∅8-0%, ∅10-0%, and ∅12-0% respectively. Even though the load was reduced following these loads, a discernible increase in the deflection was observed. During the final phase of the experiment, the maximum deflection strength for ∅8-0%, ∅10-0%, and ∅12-0% was determined to be 61.55, 56.80, and 17.92 cm, respectively. At these specific bending values, the RCB reached its maximum load-bearing capacity. It is undeniable that these findings provide substantial information regarding the capacity of RCBs to deal with loads. 

Based on the experimental findings for ∅8-5%, ∅10-5%, and ∅12-5%, it can be observed that significant bending cracks occurred in the RuCB due to the vertical stress. Figure 7 illustrates the identification of bending in RuCB under vertical stress. The occurrence of significant cracks in the RuCB is clearly indicated in Figure 8. It is possible to see in Figure 8 how the cracking of RuCBs that occurs in the bending zone is determined by the vertical load. Because of the effects of stirrups and reinforcements on the bending behavior of RuCBs, this crushing is important. At the end of this straight line, values of 33.11, 46.28, and 52.67 kN were determined for ∅8-5%, ∅10-5%, and ∅12-5%, respectively. It was found that the highest vertical load resulted in deflections of 3.96, 5.39, and 5.85 cm for ∅8-5%, ∅10-5%, and ∅12-5%, respectively. Although the load was decreased after applying these loads, there was a noticeable increase in the deflection. In the last stage of the experiment, it was found that the highest deflection for ∅8-5%, ∅10-5%, and ∅12-5% was 63.16 cm, 18.93 cm, and 14.84 cm, respectively, in that order.

The other investigations were performed for ∅8-10%, ∅10-10%, and ∅12-10%. Figure 7 shows the process of identifying deformations in the RCB when subjected to vertical loads. Figure 8 clearly demonstrates the presence of substantial cracks in the RuCB. At the end of this straight line, the values for ∅8-10%, ∅10-10%, and ∅12-10% were found to be 30.27, 40.20, and 43.83 kN, respectively. The investigation revealed that the maximum vertical load causes deflections of 4.51, 7.54, and 4.84 cm for ∅8-10%, ∅10-10%, and ∅12-10%, accordingly. Despite the reduction in load following these previous loads, a discernible increase in the degree of deflection was observed. During the last phase of the experiment, the greatest degrees of deflection for ∅8-10%, ∅10-10%, and ∅12-10% were 38.42, 12.92, and 18.29 cm, respectively.

Additional investigations were conducted for mixtures with compositions of 8–15%, 10–15%, and 12–15%. Figure 6 illustrates the deformation of the RCB upon exposure to vertical load. Figure 8 unequivocally illustrates the existence of significant fissures in RuCB. It was discovered that the values for ∅8-15%, ∅10-15%, and ∅12-15% were 28.11, 31.65, and 35.35 kN, respectively, at the conclusion of this straight line. Based on the findings of the examination, it was determined that the maximum vertical load resulted in deflections of 5.99, 4.42, and 5.34 cm for ∅8-15%, ∅10-15%, and ∅12-15%, respectively, during the investigation. Although the load was reduced after these previous loads, a noticeable increase in the deflection was observed. In the last stage of the experiment, we found that the highest levels of deflection for ∅8-15%, ∅10-15%, and ∅12-15% were 17.23, 16.63, and 21.33 cm, respectively.

### 3.2. Effects of Rubber Waste Proportions

**Figure 9:**
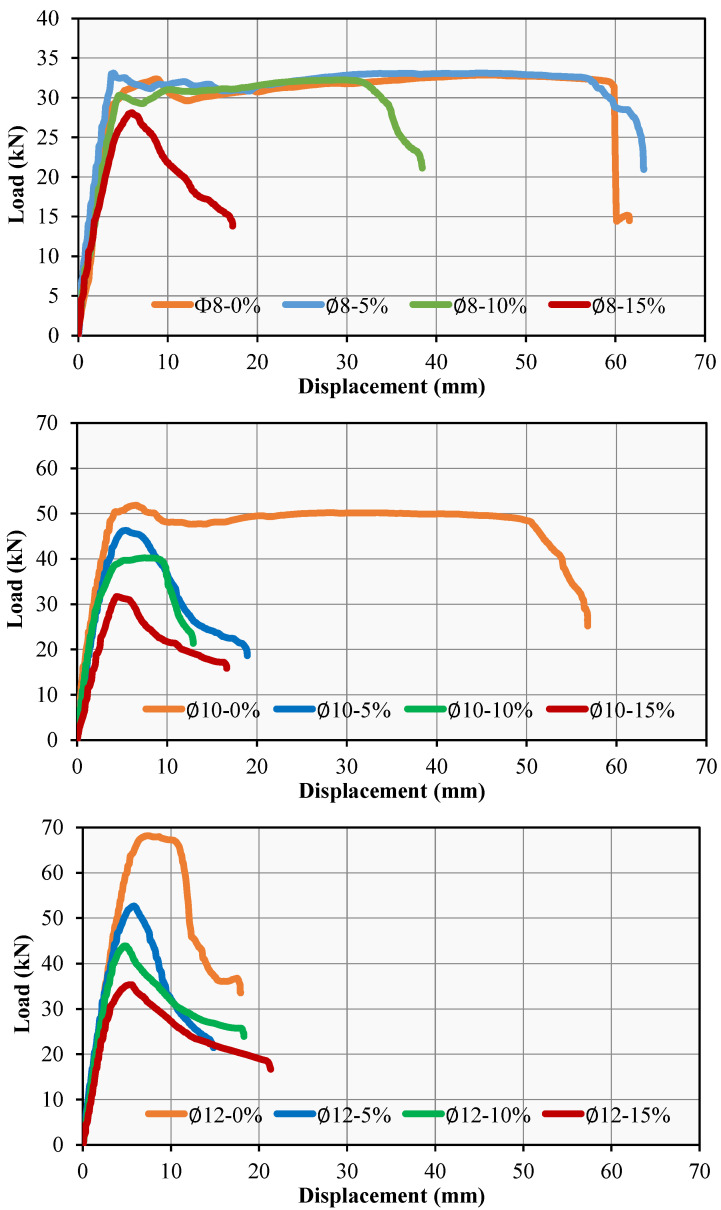
RCB load–deformation attitude.

The impact of varying the waste rubber content on the bending and fracture behaviors of RuCBs was considered. To maintain consistency, the proportions of waste rubber used in the RCBs were set to 0%, 5%, 10%, and 15%. The tension reinforcement in the RCB remained constant at 2∅8. Based on the experimental results for ∅8-0%, ∅8-5%, ∅8-10%, and ∅8-15%, it was noticed that significant bending cracks occurred in the RCB due to the vertical load, as depicted in Figure 8. Based on Figure 9, the bending increased along a straight line until reaching a specific point. The values at this point were 32.87, 33.13, 32.24, and 28.11 kN for ∅8-0%, ∅8-5%, ∅8-10%, and ∅8-15%, respectively. With a maximum vertical load of ∅8-0%, ∅8-5%, ∅8-10%, and ∅8-15%, the deflections that can be detected at that point are 8.75, 3.96, 4.52, and 5.99 cm, respectively. Following these loads, despite the reduced load, the deflection was significantly increased. After analyzing the data, it was found that at the end of the test, the maximum deflection for ∅8-0%, ∅8-5%, ∅8-10%, and ∅8-15% was 61.55 cm, 63.16 cm, 38.42 cm, and 17.23 cm, respectively, and at these deflection values, the RCB lost its ability to support the load. Based on the varying amounts of waste rubber, these results provide unquestionably significant information about the load-carrying capacity of RuCBs.

In this section of the other test, the amounts of waste rubber were varied as 0%, 5%, 10%, and 15% to examine the effects of various amounts of waste rubber on the fracture and bending attitude of RuCBs, while the tension reinforcement in the RCB was kept constant at 2∅10. Observations from the test findings of ∅10-0%, ∅10-5%, ∅10-10%, and ∅10-15% indicate that the RuCB experienced notable bending cracks under vertical stress, as shown in Figure 8. According to Figure 9, the bending exhibited a linear increase until it reached a certain threshold. The values at this specific location were 51.82, 46.29, 40.20, and 31.65 kN for ∅10-0%, ∅10-5%, ∅10-10%, and ∅10-15%, respectively. The deflection that can be observed at that point is 6.45, 5.39, 7.53, and 4.42 cm, respectively, with a maximum vertical load of ∅10-0%, ∅10-5%, ∅10-10%, and ∅10-15%. The data analysis revealed that at the end of the experiment, the maximum deflection for ∅10-0%, ∅10-5%, ∅10-10%, and ∅10-15% was 56.80 cm, 18.93 cm, 12.92 cm, and 16.63 cm, respectively. It was observed that at these deflection values, the RuCB structure lost its capacity to support the load.

While the tension reinforcement in the RuCB was kept constant at 2∅12, the amounts of waste rubber were varied as 0%, 5%, 10%, and 15% in this section of the other test to investigate the effects of different amounts of waste rubber on the fracture and bending attitude of RuCBs. The testing data of ∅12-0%, ∅12-5%, ∅12-10%, and ∅12-15% reveal that the RCB exhibited significant bending cracks when subjected to vertical stress, as depicted in Figure 8. Figure 9 shows that until it reached a particular threshold, the deflection increased linearly. The corresponding values for ∅12-0%, ∅12-5%, ∅12-10%, and ∅12-15% at this point were 68.19, 52.67, 43.83, and 35.35 kN, respectively. The measured deflections at this point were 7.37 cm, 5.85 cm, 4.84 cm, and 5.34 cm, respectively. These measurements were taken under different maximum vertical loads, specifically ∅12-0%, ∅12-5%, ∅12-10%, and ∅12-15%. According to the data analysis, the experiment concluded with the following maximum deflection measurements: 17.92 cm for ∅12-0%, 14.83 cm for ∅12-5%, 18.29 cm for ∅12-10%, and 21.33 cm for ∅12-15%. It should be noted that when subjected to these bending values, the RCB structure becomes incapable of bearing any load.

After completing these experimentally induced tests, observations were made regarding the following outcomes. Although the mechanical properties of the concrete, main/reinforcement ratio, and shear/reinforcement ratio were the same as those of the corresponding beams, it was noticed that the load capacity of the beams decreased as the inclination of the waste rubber quantity increased. There was a reduction in the amount of deformation by 37.57% and 72% when the quantity of waste rubber was increased by 10% and 15%, respectively. This is in contrast with the fact that the deformation increased by 2.63% when the waste rubber content was increased by 5%. The application of ∅8-5%, ∅8-10%, and ∅8-15% longitudinal reinforcement was the cause of this modification. A reduction in deformation of 66.67%, 77.25%, and 70.72% was associated with the application of ∅10-5%, ∅10-10%, and ∅10-15% longitudinal reinforcement, respectively. Following a reduction of 20.84% in the quantities of deformation, the application of 12-5%, 12-10%, and 12-15% longitudinal reinforcement resulted in increases of 2.02% and 15.99%, respectively.

In addition, the failure mode shifts from flexure to shear. Instead of having a negligible impact on the initial cracking load, Wu et al. [58] found that the addition of crumb rubber to reinforced concrete substantially affected the ultimate load and deflection. Prior research has noted that the results obtained were comparable to those achieved here. Furthermore, there was an increase in the number of cracks in the RuC beams when the crumb rubber content was increased from 0% to 15%, as observed in Figure 9. Because the modules of elasticity of rubber aggregates are lower than those of fine aggregates, this results in an increasing number of cracks on the beams, which eventually leads to beams colliding with themselves [59,60]. Mohamed K [59] observed an improvement in the number of cracks and a decrease in crack width as the concentration of crumb rubber rose [59]. Previous studies [59,60,61,62] have shown that the addition of crumb rubber to standard concrete results in increased cracking. This means that whether the spacing is maximized or minimized, the distance between the two cracks decreases.

### 3.3. Toughness, Stiffness, and Ductility

**Figure 10:**
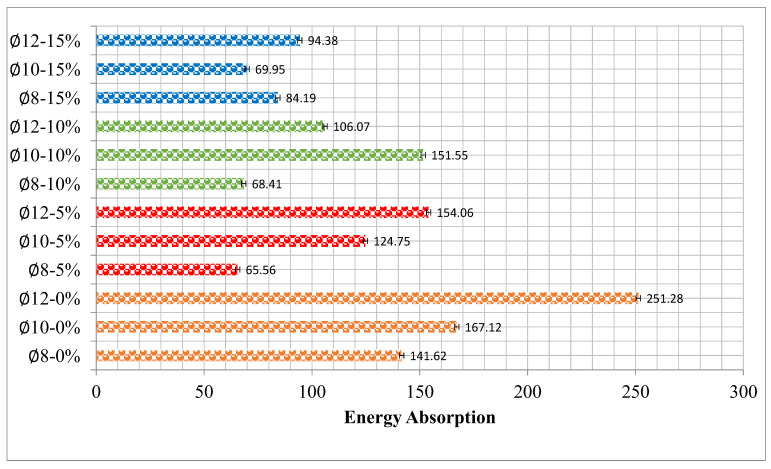
Results for total energy (toughness).

Measurements of the area encompassed by the RCB’s deflection curve during the flexural test were used to determine each mixture’s toughness (total energy), as reported in Table 4. It was found that the energy absorption capacity and ultimate load decreased as the quantity of waste rubber in the RuCB increased, as shown in Figure 10. Figure 10 demonstrates that an increase in the waste rubber content leads to a decrease in the region under the load–deflection curve. Consequently, this resulted in a loss in the energy absorption capacity of the tested beams. Although the mechanical properties of the concrete and the primary reinforcement ratio remained constant, the energy dissipation capacity of the beams decreased as the amount of waste rubber increased. Empirical evidence indicates that the energy dissipation capability of the beam decreases as the percentage of waste rubber in the beam increases. The application of longitudinal reinforcement at a percentage of ∅8-5%, ∅8-10%, and ∅8-15%, respectively, was related to a decrease in the quantities of energy dissipation by 53.71%, 51.69%, and 40.55%, respectively. With the application of ∅10-5%, ∅10-10%, and ∅10-15% longitudinal reinforcement, respectively, there was a decrease in the energy dissipation quantities by 25.35%, 9.31%, and 58.15%, respectively. Furthermore, applying ∅12-5%, ∅12-10% and ∅12-15% longitudinal reinforcement resulted in a decrease in energy dissipation quantities of 38.69%, 57.79%, and 62.44%, respectively. Generally, an increase in the ductility ratio of a structural component indicates that the part has a greater capacity to undergo significant deflections before failure. This, in turn, allows for sufficient notice before failure occurs. Gaining more tensile reinforcement often results in the sample being less ductile even if it can support a higher load. Table 4 demonstrates that an increase in the waste rubber content led to a decrease in the ductility ratio. A similarity has been observed between these findings and those published in the relevant literature. This decrease might be attributed to the concrete weakening in the compression zone at higher waste rubber percentages because of the mortar’s poor bonding with the waste rubber, which restricted the RCB’s capacity to withstand larger loads beyond the yielding point.

With the addition of waste tires, an environmentally friendly building material can be obtained, and the harmful effects of waste tires on the environment can be avoided. The positive effects on the environment are not limited to this, but they also provide an economy with the production and use of normal aggregates. 

Similarly, although waste tires reduce the compressive strength of concrete, they reduce vibrations and increase damping. Waste tires can be used in structural elements where compressive strength is not important and are subjected to tension. As can be seen from Figure 10, higher values than the toughness value obtained with the use of ∅8-0% tires were also obtained in ∅10-10% and ∅ 12-5% experiments.

However, new developments have shown that more successful mechanical results can be obtained if tires are pre-treated. For example, when tires smaller than 0.6 mm in size were used and treated with NaOH + CSBR Latex + SCA, very close compressive and flexural strength values were achieved with the reference sample even at 5%-10%-15% tire substitution values [40]. Similarly, when tires with dimensions smaller than 1.18 mm were used, compressive strength values higher than the reference specimen were obtained at a 10% tire replacement rate when cured with both methanol and acetone [41]. When 3.0 mm sized tires were used and cured with CS2, a 10% and 20% increase in RuC compressive strength was obtained at 3% and 6% tire replacement rates, respectively, compared with the reference specimens [42]. The studies carried out to improve the compressive strength of RuC were also carried out to improve its tensile strength, flexural strength, and modulus of elasticity. Research on the use of waste tires in concrete continues to develop new studies and treatment methods on the subject.

By comparing the failure load capacity with the reference RCBs, the findings demonstrated that the failure load capacity was lower. It was determined that the presence of rubber particles reduced the failure load [23]. This decrease was restricted to the presence of tiny steel fibers. This is because the rubber particles are fragile and compressed, and the loading capacity decreases because of its influence on the strength of other components of the concrete. The failure load capacity was significantly affected by the rubber combinations, which also contributed to the increase in the blanks. The reduction was limited by the lack of tiny steel fibers [23].

## 4. Conclusions

This study aimed to evaluate the effectiveness of using waste rubber as a replacement for parts of aggregates to improve the sustainability of concrete. With the aim of achieving this goal, a total of 12 specimens were produced, and to evaluate the bending behavior of these specimens, several experiments were carried out. Several selected parameters were the effects of the waste rubber ratio and the longitudinal reinforcement ratio. Using replacement rates of 0%, 5%, 10%, and 15%, the total number of aggregates was altered to achieve this objective. The longitudinal reinforcement consisted of three diameters: ∅6 at the top (for every beam), and ∅8, ∅10, and ∅12 at the bottom. Several important facts are evaluated in the following analysis:

- Based on the slump test results, an increase in the quantity of waste rubber in the concrete mixture led to a decrease in the slump value of the concrete. The increase in the proportion of waste rubber from 0% to 15% resulted in an associated decrease in CS after 28 days (34.17%, 57.74%, and 67.52%). Furthermore, the split tensile strength and flexural strength decreased by 24.15%, 38.0%, and 44.49%, and 24.92%, 49.12%, and 54.84%, respectively, when the proportion of waste rubber increased from 0% to 5%, 10%, and 15%, respectively.
- Based on the experimental results, it was found that as the percentage of waste rubber in the concrete mixture improved, the maximum load-carrying value in the RCBs decreased. After comparing RCBs with several levels of waste rubber, we found that the RCBs with ∅8-5% and waste rubber exhibited the highest bending in the center.
- This research thoroughly examines the impact of varying tension reinforcement levels on the crack formation and bending characteristics of RCBs. Based on the experimental findings, it was noticed that using the complete quantity of waste rubber in the RCBs resulted in an increase in the bending strength of the RCBs that used ∅12 tensile reinforcement compared to the other samples.
- It was found that increasing the quantity of waste rubber in the RCB reduced the energy absorption capacity and ultimate load of the RCB. The application of longitudinal reinforcement at 5%, 10%, and 15% correspondingly resulted in a decrease in energy dissipation for ∅8 by 53.71%, 51.69%, and 40.55%, respectively. In addition, there were decreases of 38.69%, 57.79%, and 62.44% for ∅12, and 25.35%, 9.31%, and 58.15% for ∅10.

### Recommendations for Further Investigation

In this study, only a fiber-shaped waste tire was used. Bending and shear investigations in reinforced-concrete beams can be carried out by using the crumbs from waste rubber and crumb-fiber-form waste rubber together. In addition, the bending and shear behaviors of beams can be investigated after pre-treating the rubber to increase the bond between the rubber and cement paste.

## Figures and Tables

**Table 1:** Blend ratios.

	Coarse Aggregate (kg)				Fine Aggregate (kg)			Cement (C) kg/m^3^	Water (W) kg/m^3^	W/C	Super Plas. kg	Unit Weight kg/m^3^
#	4–11.2 mm	11.2–22.4 mm	Rubber (%)	Rubber (kg)	0–4 mm	Rubber (%)	Rubber (kg)					
RNC	328.15	483.59	0.00	0.00	1107.26	0.00	0.00	270	186.30	0.69	1.080	2376
RuNC%5	311.74	459.41	5	16.27	1051.90	5	19.78	270	186.30	0.69	1.080	2316
RuNC%10	295.34	435.23	10	32.53	996.54	10	39.56	270	186.30	0.69	1.080	2257
RuNC%15	278.93	411.05	15	48.80	941.17	15	59.34	270	186.30	0.69	1.080	2197

**Table 2:** Characteristics of cement.

Chemical Properties	Obtained Values	TS EN 197-1	
		Least	Most
Loss of Glow (%)	4.36	-	5.00
Insoluble Residue (%)	0.50	-	5.00
Sulfur Trioxide (SO_3_) (%)	3.53	-	4.00
Chloride (Cl) (%)	0.021	-	0.10
K_2_O (%)	0.64	-	-
Na_2_O (%)	0.32	-	-
Physical Properties	Obtained values	TS EN 197-1	
		Least	Most
Specific Surface (${cm}^{2}$/g)	3758	-	-
Two-day CS (MPa)	25.58	20	-
Twenty-eight-day CS (MPa)	47.1	42.5	62.5
Initial Setting (min.)	161	60	-
Volume Expansion (mm)	2	-	10

**Table 3:** Characteristics of the specimen.

Name	Specimen	Long. Reinf.	Sti. Dia./Spac	Stirrup Vol. Ratio (δ_w_)	W_ru_ (%)
RNC∅8	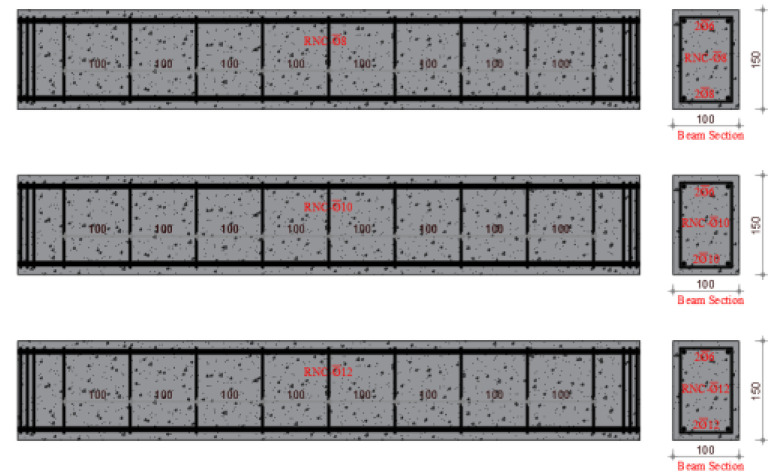	2∅6 (top) 2∅8 (bot.)	∅6/10	0.0057	0
RNC∅10		2∅6 (top) 2∅10 (bot.)			0
RNC∅12		2∅6 (top) 2∅12 (bot.)			0
RuNC∅8-%5	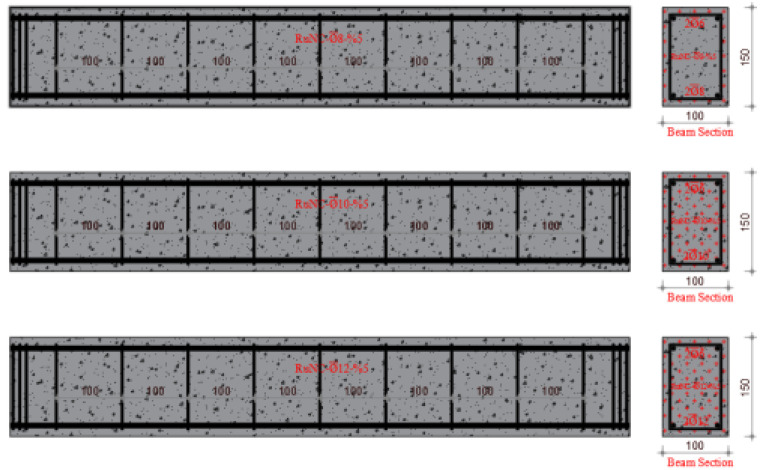	2∅6 (top) 2∅8 (bot.)	∅6/10	0.0057	5
RuNC∅10-%5		2∅6 (top) 2∅10 (bot.)			5
RuNC∅12-%5		2∅6 (top) 2∅12 (bot.)			5
RuNC∅8-%10	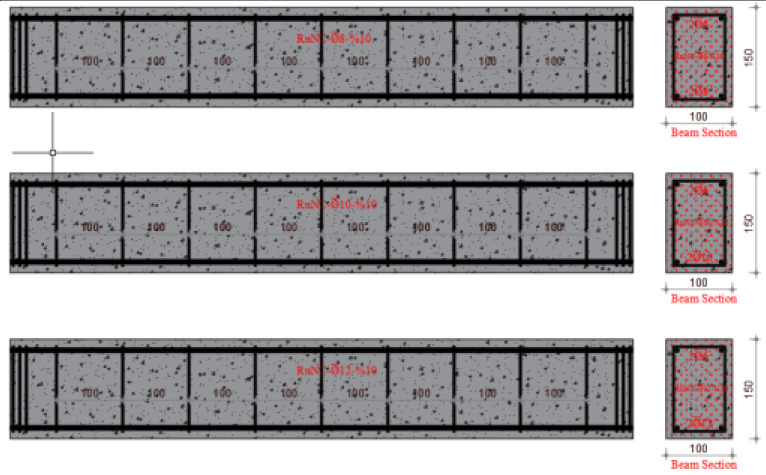	2∅6 (top) 2∅8 (bot.)	∅6/10	0.0057	10
RuNC∅10-%10		2∅6 (top) 2∅10 (bot.)			10
RuNC∅12-%10		2∅6 (top) 2∅12 (bot.)			10
RuNC∅8-%15	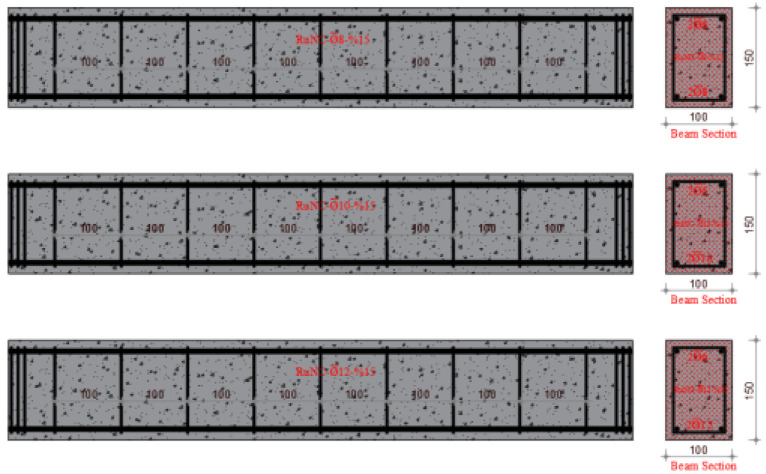	2∅6 (top) 2∅8 (bot.)	∅6/10	0.0057	15
RuNC∅10-%15		2∅6 (top) 2∅10 (bot.)			15
RuNC∅12-%15		2∅6 (top) 2∅12 (bot.)			15

**Table 4:** Significance of tests on load and displacement values.

Test No	P_Max_ (kN)	Def. at P_Max_ (mm)	*δ_u_* (mm)	Stiffness at Pmax(kN/mm)	*P_u_* (0.85*P_max_*) (kN)	Displacement at Yield *δ_y_* (mm)	Stiffness at Yield (kN/mm)	Ductility Ratio
∅8-%0	32.37	8.75	61.54	3.70	27.51	3.27	8.41	18.81
∅10-%0	51.82	6.45	56.80	8.03	44.05	3.25	13.55	17.47
∅12-%0	68.19	7.37	17.92	9.25	57.96	4.70	12.33	3.81
∅8-%5	33.11	3.96	63.16	8.36	28.14	3.04	9.25	20.77
∅10-%5	46.29	5.39	18.93	8.59	39.35	3.18	12.37	5.95
∅12-%5	52.67	5.85	14.83	9.00	44.77	3.73	12.00	3.97
∅8-%10	30.27	4.52	38.42	6.70	25.73	3.50	7.35	10.97
∅10-%10	40.20	7.54	12.92	5.33	34.11	3.10	11.00	4.17
∅12-%10	43.83	4.84	18.29	9.06	37.26	3.13	11.90	5.84
∅8-%15	28.11	5.99	17.23	4.69	23.89	3.78	6.32	4.57
∅10-%15	31.65	4.42	16.63	7.16	26.90	3.36	8.00	4.95
∅12-%15	35.35	5.34	21.33	6.62	30.05	3.13	9.60	6.81

## Data Availability

The original contributions presented in the study are included in the article, further inquiries can be directed to the corresponding author.

## References

[1] Azunna S.U., Aziz F.N., Rashid R.S., Bakar N.B. (2024). Review on the characteristic properties of crumb rubber concrete. Clean. Mater..

[2] Yıldızel S.A., Özkılıç Y.O., Bahrami A., Aksoylu C., Başaran B., Hakamy A., Arslan M.H. (2023). Experimental investigation and analytical prediction of flexural behaviour of reinforced concrete beams with steel fibres extracted from waste tyres. Case Stud. Constr. Mater..

[3] Torretta V., Rada E.C., Ragazzi M., Trulli E., Istrate I.A., Cioca L.I. (2015). Treatment and disposal of tyres: Two EU approaches. A review. Waste Manag..

[4] (2024). MSV Mediaservice & Verlag GmbH. https://global-recycling.info/.

[5] Xiong Z., Li H., Pan Z., Li X., Lu L., He M., Li H., Liu F., Feng P., Li L. (2024). Fracture properties and mechanisms of steel fiber and glass fiber reinforced rubberized concrete. J. Build. Eng..

[6] Lin Q., Liu Z., Sun J., Yu L. (2023). Comprehensive modification of emulsified asphalt on improving mechanical properties of crumb rubber concrete. Constr. Build. Mater..

[7] Ma Q., Mao Z., Lei M., Zhang J., Luo Z., Li S., Du G., Li Y. (2023). Experimental investigation of concrete prepared with waste rubber and waste glass. Ceram. Int..

[8] Roychand R., Gravina R.J., Zhuge Y., Ma X., Youssf O., Mills J.E. (2020). A comprehensive review on the mechanical properties of waste tire rubber concrete. Constr. Build. Mater..

[9] Yildizel S.A., Özkılıç Y.O., Yavuz A. (2024). Optimization of waste tyre steel fiber and rubber added foam concretes using Taguchi method and artificial neural networks. Structures.

[10] Gheni A.A., Alghazali H.H., ElGawady M.A., Myers J.J., Feys D. (2019). Durability properties of cleaner cement mortar with by-products of tire recycling. J. Clean. Prod..

[11] Topcu I.B. (1995). The properties of rubberized concretes. Cem. Concr. Res..

[12] Chung K.H., Hong Y.K. (2009). Weathering properties of elastic rubber concrete comprising waste tire solution. Polym. Eng. Sci..

[13] Aslani F. (2016). Mechanical properties of waste tire rubber concrete. J. Mater. Civ. Eng..

[14] Reda Taha M.M., El-Dieb A.S., Abd El-Wahab M., Abdel-Hameed M. (2008). Mechanical, fracture, and microstructural investigations of rubber concrete. J. Mater. Civ. Eng..

[15] Akter M., Sulong N.R., Ayough P., Tafsirojjaman T., Fawzia S. (2024). Flexural behavior of circular rubberized concrete-filled double-skin steel tubular beams: Experiments. Eng. Struct..

[16] Toutanji H.A. (1996). The use of rubber tire particles in concrete to replace mineral aggregates. Cem. Concr. Compos..

[17] Dhivya K., Priyadharshini K. (2022). Experimental study on strength properties of concrete with partial replacement of coarse aggregate by rubber tyre waste. Mater. Today Proc..

[18] Etli S. (2023). Evaluation of the effect of silica fume on the fresh, mechanical and durability properties of self-compacting concrete produced by using waste rubber as fine aggregate. J. Clean. Prod..

[19] Abdelmonem A., El-Feky M., Nasr E.-S.A., Kohail M. (2019). Performance of high strength concrete containing recycled rubber. Constr. Build. Mater..

[20] Karunarathna S., Linforth S., Kashani A., Liu X., Ngo T. (2021). Effect of recycled rubber aggregate size on fracture and other mechanical properties of structural concrete. J. Clean. Prod..

[21] Chou L.H., Lu C.-K., Chang J.-R., Lee M.T. (2007). Use of waste rubber as concrete additive. Waste Manag. Res..

[22] Han X., Zhou S., Chen A., Feng L., Ji Y., Wang Z., Sun S., Li K., Xia X., Zhang Q. (2024). Analytical evaluation of stress–strain behavior of rubberized concrete incorporating waste tire crumb rubber. J. Clean. Prod..

[23] Mohammed H.H., Ali A.S. (2023). Flexural behavior of reinforced rubberized reactive powder concrete beams under repeated loads. J. Eng..

[24] Hossain F.Z., Pal A., Ahmed K.S., Bediwy A., Alam M.S. (2023). Shear behavior of polypropylene fiber-reinforced concrete beams containing recycled aggregate and crumb rubber. J. Clean. Prod..

[25] AL-Hajjar K.R., AL-Khafaji A.G. (2024). Behavior of rubberized reinforced concrete beams under impact loading. Proceedings of AIP Conference Proceedings.

[26] Kadhim A.A., Kadhim H.M. (2023). Experimental investigation of rubberized reinforced concrete continuous deep beams. J. King Saud Univ. Eng. Sci..

[27] Jayanath A., Gamage J., Chandrathilaka E. (2023). Flexural Behaviour of Reinforced Rubberised Concrete Beam.

[28] Sharaky I., Seleem M., Elamary A.S. (2023). Minimizing the crumb rubber effects on the flexural behaviour of the layered RC beams cast using rubberized concrete with or without recycled tire steel fibers. Constr. Build. Mater..

[29] Karalar M., Ozturk H., Ozkilic Y.O. (2023). Experimental and numerical investigation on flexural response of reinforced rubberized concrete beams using waste tire rubber. Steel Compos. Struct..

[30] Eisa A.S., Elshazli M.T., Nawar M.T. (2020). Experimental investigation on the effect of using crumb rubber and steel fibers on the structural behavior of reinforced concrete beams. Constr. Build. Mater..

[31] Alasmari H.A., Bakar B., Akil H. (2020). Influence of rubberized-fibrous concrete on flexural behavior of hybrid reinforced beam. Proceedings of AIP Conference Proceedings.

[32] Hasan T.M., Ali A.S. (2020). Flexural behavior of fiber reinforced self-compacting rubberized concrete beams. J. Eng..

[33] Ferdous W., Manalo A., AlAjarmeh O.S., Zhuge Y., Mohammed A.A., Bai Y., Aravinthan T., Schubel P. (2021). Bending and shear behaviour of waste rubber concrete-filled FRP tubes with external flanges. Polymers.

[34] Gurung S. (2020). Flexural Analysis of Rubberized Concrete Beams Using Finite Element Method. https://www.theseus.fi/handle/10024/338913.

[35] AbdelAleem B.H., Hassan A.A. (2022). Use of rubberized engineered cementitious composite in strengthening flexural concrete beams. Eng. Struct..

[36] Nematzadeh M., Mousavi R. (2021). Post-fire flexural behavior of functionally graded fiber-reinforced concrete containing rubber. Comput. Concr..

[37] Tang Y., Feng W., Chen Z., Nong Y., Guan S., Sun J. (2021). Fracture behavior of a sustainable material: Recycled concrete with waste crumb rubber subjected to elevated temperatures. J. Clean. Prod..

[38] Shahjalal M., Islam K., Rahman J., Ahmed K.S., Karim M.R., Billah A.M. (2021). Flexural response of fiber reinforced concrete beams with waste tires rubber and recycled aggregate. J. Clean. Prod..

[39] Nawar M.T., Eisa A.S., Elshazli M.T., Ibrahim Y.E., El-Zohairy A. (2024). Numerical Analysis of Rubberized Steel Fiber Reinforced Concrete Beams Subjected to Static and Blast Loadings. Infrastructures.

[40] Li G., Wang Z., Leung C.K., Tang S., Pan J., Huang W., Chen E. (2016). Properties of rubberized concrete modified by using silane coupling agent and carboxylated SBR. J. Clean. Prod..

[41] Rivas-Vázquez L., Suárez-Orduña R., Hernández-Torres J., Aquino-Bolaños E. (2015). Effect of the surface treatment of recycled rubber on the mechanical strength of composite concrete/rubber. Mater. Struct..

[42] Emam E., Yehia S. (2018). Experimental study on enhanced crumb rubber concrete. Int. J. Sci. Eng. Res.

[43] Najim K.B., Hall M.R. (2013). Crumb rubber aggregate coatings/pre-treatments and their effects on interfacial bonding, air entrapment and fracture toughness in self-compacting rubberised concrete (SCRC). Mater. Struct..

[44] Segre N., Joekes I. (2000). Use of tire rubber particles as addition to cement paste. Cem. Concr. Res..

[45] Muñoz-Sánchez B., Arévalo-Caballero M.J., Pacheco-Menor M.C. (2017). Influence of acetic acid and calcium hydroxide treatments of rubber waste on the properties of rubberized mortars. Mater. Struct..

[46] Ecemis A.S., Madenci E., Karalar M., Fayed S., Althaqafi E., OzkillC Y.O. (2024). Shear performance of reinforced concrete beams with rubber as form of fiber from waste tire. Steel Compos. Struct..

[47] Yung W.H., Yung L.C., Hua L.H. (2013). A study of the durability properties of waste tire rubber applied to self-compacting concrete. Constr. Build. Mater..

[48] Turatsinze A., Garros M. (2008). On the modulus of elasticity and strain capacity of self-compacting concrete incorporating rubber aggregates. Resour. Conserv. Recycl..

[49] (2012). Cement—Part 1: Composition, Specifications and Conformity Criteria For Common Cements.

[50] (1916). Standard Specification for Ready Mixed Concrete.

[51] (2011). Admixtures for Concrete, Mortar and Grout—Part 2: Concrete Admixtures; Definitions, Requirements, Conformity, Marking and Labelling.

[52] Scrivener K.L., Crumbie A.K., Laugesen P. (2004). The interfacial transition zone (ITZ) between cement paste and aggregate in concrete. Interface Sci..

[53] Neville A.M. (1995). Properties of Concrete.

[54] Ismail M.K., Hassan A.A.A. (2017). Shear behaviour of large-scale rubberized concrete beams reinforced with steel fibres. Constr. Build. Mater..

[55] Najim K., Hall M. (2010). A review of the fresh/hardened properties and applications for plain-(PRC) and self-compacting rubberised concrete (SCRC). Constr. Build. Mater..

[56] Alasmari H.A., Bakar B., Noaman A. (2019). A comparative study on the flexural behaviour of rubberized and hybrid rubberized reinforced concrete beams. Civ. Eng. J..

[57] Mohammed B.S., Hossain K.M.A., Swee J.T.E., Wong G., Abdullahi M. (2012). Properties of crumb rubber hollow concrete block. J. Clean. Prod..

[58] Wu Z., Shi C., He W., Wu L. (2016). Effects of steel fiber content and shape on mechanical properties of ultra high performance concrete. Constr. Build. Mater..

[59] Ismail M.K., Hassan A.A. (2017). An experimental study on flexural behaviour of large-scale concrete beams incorporating crumb rubber and steel fibres. Eng. Struct..

[60] Ismail M.K., Hassan A.A. (2017). Ductility and cracking behavior of reinforced self-consolidating rubberized concrete beams. J. Mater. Civ. Eng..

[61] Noaman A.T., Bakar B.A., Akil H.M., Alani A. (2017). Fracture characteristics of plain and steel fibre reinforced rubberized concrete. Constr. Build. Mater..

[62] Ismail M.K., Hassan A.A., Hussein A.A. (2017). Structural behaviour of reinforced concrete beams containing crumb rubber and steel fibres. Mag. Concr. Res..

